# Quantitative CT Variables Enabling Response Prediction in Neoadjuvant Therapy with EGFR-TKIs: Are They Different from Those in Neoadjuvant Concurrent Chemoradiotherapy?

**DOI:** 10.1371/journal.pone.0088598

**Published:** 2014-02-26

**Authors:** Yousun Chong, Jae-Hun Kim, Ho Yun Lee, Yong Chan Ahn, Kyung Soo Lee, Myung-Ju Ahn, Jhingook Kim, Young Mog Shim, Joungho Han, Yoon-La Choi

**Affiliations:** 1 Department of Radiology and Center for Imaging Science, Sungkyunkwan University School of Medicine, Seoul, Korea; 2 Department of Radiation Oncology, Sungkyunkwan University School of Medicine, Seoul, Korea; 3 Division of Hemato-Oncology, Department of Internal Medicine, Sungkyunkwan University School of Medicine, Seoul, Korea; 4 Department of Thoracic Surgery, Sungkyunkwan University School of Medicine, Seoul, Korea; 5 Department of Pathology, Samsung Medical Center, Sungkyunkwan University School of Medicine, Seoul, Korea; Memorial Sloan-Kettering Cancer Center, United States of America

## Abstract

**Background and Purpose:**

To correlate changes of various CT parameters after the neoadjuvant treatment in patients with lung adenocarcinoma with pathologic responses, focused on their relationship with different therapeutic options, particularly of EGFR-TKI and concurrent chemoradiation therapy (CCRT) settings.

**Materials and Methods:**

We reviewed pre-operative CT images of primary tumors and surgical specimens obtained after neoadjuvant therapy (TKI, n = 23; CCRT, n = 28) from 51 patients with lung adenocarcinoma. Serial changes in tumor volume, density, mass, skewness/kurtosis, and size-zone variability/intensity variability) were assessed from CT datasets. The changes in CT parameters were correlated with histopathologic responses, and the relationship between CT variables and histopathologic responses was compared between TKI and CCRT groups.

**Results:**

Tumor volume, mass, kurtosis, and skewness were significant predictors of pathologic response in CCRT group in univariate analysis. Using multivariate analysis, kurtosis was found to be independent predictor. In TKI group, intensity variability and size-zone variability were significantly decreased in pathologic responder group. Intensity variability was found to be an independent predictor for pathologic response on multivariate analysis.

**Conclusions:**

Quantitative CT variables including histogram or texture analysis have potential as a predictive tool for response evaluation, and it may better reflect treatment response than standard response criteria based on size changes.

## Introduction

Locally advanced non-small cell lung cancer (NSCLC) has a dismal prognosis with a median overall survival (OS) of 25–35 months despite multimodal treatment including radiation therapy (RT), chemotherapy and surgery [1], [2]. Induction concurrent chemoradiation therapy (CCRT) is known to result in short-term gross tumor volume reduction, with aggressive locoregional control. Previous studies demonstrated variable responses with volume reduction averaging 38% to 73% [3], with improved survival compared with the treatment of surgery alone [4]. This may be explained by the potent local control effect of irradiation. Therefore, there is a need for studies directed toward predicting treatment benefit versus risk of treatment failure. Clinically, such predictors would allow further individualization of treatment during radiotherapy [5].

On the other hand, during the last decade, several molecular-targeted agents—for example, epidermal growth factor receptor tyrosine kinase inhibitors (EGFR-TKIs) such as erlotinib and gefitinib—have emerged for treatment of NSCLC [6], as well as in the neoadjuvant setting, which has also shown to be effective in a subset of patients with NSCLC [7]–[10]. Furthermore, although TKI agents are most active in patients with an EGFR mutation, patients without documented mutation still showed survival benefit compared with placebo [9].

With the advancement in imaging techniques and their increasing application to oncology practice, imaging-based tumor volume regression rate evaluated at mid-RT has been shown to predict local control rate and disease-free survival (DFS) after RT or CCRT [11], [12]. In addition, quantitative measurement of tumor regression rate becomes more realistic with the use of imaging — particularly during therapy when the morphologic changes remain subtle and difficult to assess by clinical examination [12], [13]. Evaluation of treatment response to TKI agents is also challenging. It is well established that the conventional RECIST underestimates response rates than the proportion of patients who actually experience clinically effective disease control [7], [10], [14]–[20]. Since TKI agents aim for inhibition of tumor cell growth, but not necessarily tumor cell death, tumor response may not emerge as early decrease in tumor size [19]. Very recently, histogram analysis or texture analysis is receiving attention as a method for quantifying tumor heterogeneity and evaluating treatment response [14], [21], [22].

Here, the question remains which is more effective to predict treatment response in the various imaging-based quantitative assessment methods, and whether or not each method acts differently depending on therapeutic regimen.

Given the need for clinical validation of any updated analysis tool, our main objectives were to identify differences in serial changes of various CT-based parameters in patients with lung adenocarcinoma scheduled to undergo surgical resection after neoadjuvant therapy, and to correlate those changes with pathologic responses, focused on their relationship with different neoadjuvant therapeutic options, particularly of EGFR-TKI and concurrent chemoradiation therapy (CCRT) settings.

## Materials and Methods

The institutional review board of Samsung Medical Center (SMC IRB) approved this retrospective study with a waiver of informed consent.

### Patients

From September 2005 through December 2011, 398 patients with stage IIIA NSCLC underwent curative surgical resection of lung cancer at our institution, after neoadjuvant treatment (chemotherapy, radiation therapy, or both). Among these patients, patients with pathologically proven adenocarcinoma were only included, while other histologic subtypes (such as squamous cell carcinoma, large cell carcinoma, small cell lung cancer, neuroendocrine cancer, etc.) were excluded. We subdivided the patients into three groups, depending on different neoadjuvant treatment options: chemotherapy with TKI agents, chemotherapy with conventional agents, and CCRT. Since our interest lay in comparing the imaging parameter changes for treatment response prediction between novel TKI and CCRT as a neoadjuvant option, patients who underwent neoadjuvant chemotherapy with conventional agents were excluded. As a result, two groups of patients were enrolled in our study: patients who underwent neoadjuvant chemotherapy with TKI agents, and those who underwent CCRT. Patients for neoadjuvant TKI agent were assembled from a selected population fulfilling ≥ two of the following features: female, adenocarcinoma, nonsmoker, and Asian. Regimen of TKI group comprised 1 tablet of 150 mg of erlotinib daily for 3 weeks. Neoadjuvant CCRT included chemotherapy and concurrent thoracic radiotherapy (TRT). The chemotherapy regimen was weekly paclitaxel (50 mg/m^2^ per week IV) plus cisplatin (25 mg/m^2^ per week IV) or weekly paclitaxel (50 mg/m^2^ per week IV) plus carboplatin (AUC 1.5/week IV) for 5 weeks. The concurrent TRT dose was 45 Gy over 5 weeks (1.8 Gy/fraction per day, 5 fractions/week). Surgical resection was planned around 6 weeks after the completion of neoadjuvant therapy for CCRT group, and was scheduled in the fourth week after start of treatment for TKI group. Surgical resection involved a radical resection of the tumor, preferably by lobectomy, and regional lymph node dissection.

Ultimately, 51 patients (25 men and 26 women) satisfied our inclusion criteria. Twenty-three patients received neoadjuvant chemotherapy with TKI, while 28 patients underwent concurrent chemoradiation therapy.

### Imaging protocol

In all patients, baseline contrast-enhanced CT before treatment commencement, and follow up contrast-enhanced CT after 4–6 weeks of neoadjuvant treatment were performed.

### Image data analysis

A thoracic radiologist (Y.C., with 4 years of experience in thoracic CT interpretation) who was unaware of other patient data evaluated the acquired images semiquantitatively. Only the primary tumors were analyzed. Tumors were segmented by drawing a region of interest (ROI) covering as large an area as possible of the whole tumor. Next, voxel-based CT numbers were collected from lesion segmentations. Since the lesions were segmented to cover the entire tumor, larger lesions had more number of segments than smaller lesions.

**Figure 1:**
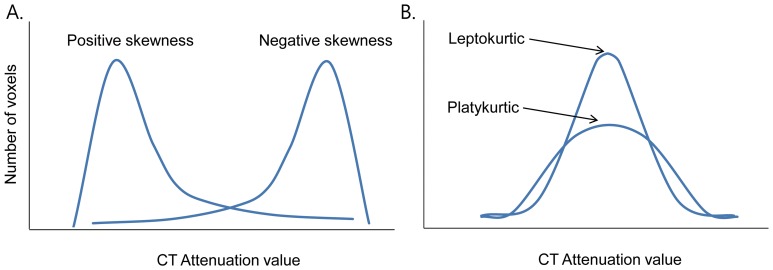
Histogram distributions of CT attenuation value. (a) Skewness. A negative skewness indicates an elongated tail on the left side of the mean, with most values lying to the right of the mean. A positive skewness indicates an elongated tail on the right side of the mean, with most values lying to the left of the mean. (b) Kurtosis. Leptokurtosis indicates a sharper peak, and platykurtosis indicates a flatter peak.

For tumor density and volume, the computer automatically calculated the density (g/cm^3^) from mean attenuation of total voxels and volume (cm^3^) by multiplying the number of voxels by the unit volume of a voxel [23]. Tumor mass (in grams) was calculated by multiplying tumor volume (in cubic centimeters) by mean tumor density [24]. Next, a spreadsheet of all of the values was created, which was used to compute histogram distribution parameters of kurtosis and skewness [25]. The skewness and kurtosis were computed from the segmented tumor region. On histograms, skewness represented the distribution pattern of CT attenuation values; negative and positive skewness indicated that the data were more spread to the left and right of the mean, respectively. Kurtosis represented the position of peak height that indicates CT attenuation value of the maximum number of voxels, with leptokurtic indicated by a sharper peak and platykurtic indicated by a flatter peak (Figure 1).

Texture analysis was performed by a radiology physicist (J.H.K.) with four years of experience in radiology physics. Voxel values within the segmented tumors were resampled to yield 16 of discrete values in order to reduce the noise in image and to normalize the intensity across subjects by clustering voxels with similar intensities [26]. From the discrete tumor images (16 gray levels), the gray level size zone matrix was computed. The value of the matrix’s (m, n) is defined by the number of homogenous regions given the homogeneous tumor size (n) to their intensity (m). For example, ‘Matrix’s (3, 5) is 6.’ means that there are 6 homogenous regions, in which the grey level of each cluster is 3, and the size of each cluster is 5 voxels. This gray level size zone matrix was used to compute the variability in the size and the intensity of homogeneous tumor regions [26], [27].

### Pathologic evaluation of treatment response

Pathologic response was used as the reference standard of therapeutic response. An experienced lung pathologist (J.H. with 20 years of experience in lung pathology) retrospectively interpreted entire tissue sections sliced at 5- to 10-mm intervals and measured the proportion (%) of viable tumor cells in the primary tumor of the resected surgical specimens [28]. Tumor regression was scored as ’pathologic response’ if more than 50% necrosis was present with morphologic signs of therapy-induced regression or ‘no response’ when either 0%–50% necrosis or necrosis that could not be attributed to the therapy effect was seen [6]. If more than 90% necrosis was present in the resected specimen, tumor regression was defined as ‘near- complete pathologic response’ [6].

In addition, comprehensive histologic subtyping was made for the primary tumor in a semi-quantitative manner. Tumors were stratified into the following three grades based on histologically predominant subtypes as: low grade; intermediate grade; and high grade [29], [30].

*EGFR/KRAS* mutational status had been determined by a pathologist (Y.L.C.) by using polymerase chain reaction (PCR) and a direct DNA sequencing method, as previously described [31].

### Statistical analysis

Statistical differences between TKI group and CCRT group were analyzed by using either the Mann Whitney test or the Fisher exact test. To identify variables that could be used in differentiating pathologic responders from nonresponders, logistic regression analysis was conducted. Characteristics with a *p* value of less than 0.50 at univariate analysis were used as the input variables for multiple logistic regression analysis. In multiple logistic regression analysis, a backward stepwise selection mode was used, with iterative entry of variables on the basis of test results (*p* < 0.05). The removal of variables was based on likelihood ratio statistics with a probability of 0.10. ROC analysis was also performed to evaluate the differentiating performance of multiple logistic regression models in discriminating pathologic responders from nonresponders. All analysis was performed using SPSS for Windows, version 12.0 (SPSS, Inc., Chicago, IL).

## Results

### Baseline characteristics

The TKI group consisted of 23 patients, and the CCRT group included 28 patients. There was a discrepancy in gender distribution and smoking habit, with female predominance(17 out of 23) in the TKI group and male predominance(19 out of 28) in the CCRT group (*p* = 0.004). This was because never smoker females were selected and included in the neoadjuvant TKI treatment planning. Otherwise, all baseline CT and PET features did not show significant difference between the two groups (Table 1). Also, all pathologic and genetic features did not differ significantly in each group.

**Table 1:** Clinicopathologic and baseline radiologic characteristics according to the neoadjuvant regimen.

	TKI group	CCRT group	
	(n = 23)	(n = 28)	*P* values
**Age (y)** **	55.6±9.1	56.5±10.5	.732
**Gender**			**.004** *
Male	6 (26)	19 (68)	
Female	17 (74)	9 (32)	
**Smoking habit**			**<.001** *
Never	22 (96)	14 (50)	
Ever	1 (4)	14 (50)	
**Histology type**			
Adenocarcinoma	23 (100)	28 (100)	1.00
**Stage**			
IIIA	23 (100)	28 (100)	1.00
**Baseline CT features**			
Volume (cm^3^)	22.7±23.8	31.9±47.8	.405
Density	1.13±0.51	1.14±0.34	.458
Mass (g)	25.9±28.1	36.6±55.2	.403
Histogram Analysis			
Kurtosis	35.5±87.5	17.2±11.9	.280
Skewness	–1.91±4.58	–2.42±1.99	.600
Texture Analysis			
Intensity variability	8.00±3.83	6.87± 4.42	.338
Size-zone variability	23.5±10.8	22.7±18.6	.857
**Baseline PET features**			
SUVmax**	10.3±3.8	12.7±6.5	.180
**Pathologic features**			
Response			.924
Responder+	10 (43)	12 (43)	
Near complete responder++	4 (17)	7 (25)	
Nonresponder	13 (57)	16 (57)	
Differentiation			.066
Well-	1 (4)	0 (0)	
Moderately-	21 (92)	21 (75)	
Poorly-	1 (4)	7 (25)	
***EGFR*** ** mutation**			.721
Positive	11 (48)	5 (18)	
Negative	10 (43)	6 (21)	
Unknown	2 (9)	17 (61)	
***KRAS*** ** mutation**			.467
Positive	0 (0)	2 (7)	
Negative	7 (30)	6 (21)	
Unknown	16 (70)	20 (72)	

TKI, tyrosine kinase inhibitors; CCRT, concurrent chemoradiation therapy.

Note.—Unless otherwise indicated, data in parentheses are percentages.

* *P* <.05.

** Data are the range.

+ Less than 50% of viable tumor cells in the resected specimen.

++ Less than 10% of viable tumor cells in the resected specimen.

### CT parameters and pathologic response

**Figure 2:**
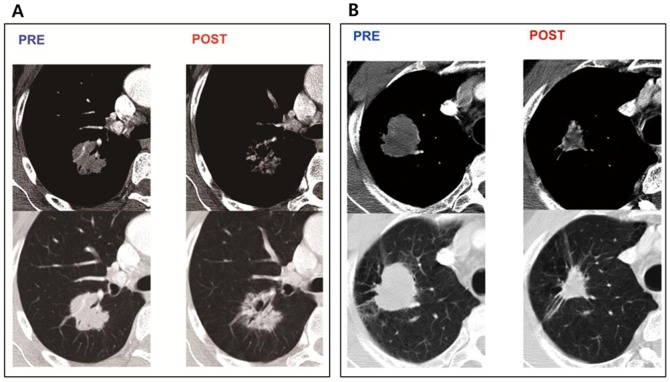
Quantitative CT parameters of pathologic responder. (a) Combined histogram distributions of the treatment before (blue) and after (red). As for histogram distribution, the vertical axis in each histogram shows the number of pixels in the segmented tumor. The horizontal axis shows the CT attenuation values. Attenuation value is shifted to the left from the blue to the red graph. (b) Intensity size-zone matrix of the treatment before (blue) and after (red). The horizontal axis shows size of homogenous area. Divergent distribution on the horizontal axis indicates increased size-zone variability. The vertical axis shows intensity. Divergent distribution on the vertical axis indicates increased intensity variability. Distributions of the points on the matrix become more convergent and clustered both in the horizontal axis and the vertical axis from the pre-treatment matrix to the post-treatment matrix.

Percent changes of CT parameters in pathologic responders and pathologic nonresponders were tabulated and compared in both CCRT group and TKI group (Table 2, Figure 2). In case of CCRT group, percent decreases of tumor volume and mass were significantly greater in pathologic responders, as compared with pathologic nonresponders (*p*s = 0.028 and 0.018, respectively).

**Table 2:** Percent changes of CT parameters in relation to pathologic response.

	TKI group (n = 23)			CCRT group (n = 28)		
	Pathologic responder	Pathologic nonresponder		Pathologic responder	Pathologic nonresponder	
CT parameters	(n = 10)	(n = 13)	*P* values	(n = 12)	(n = 16)	*P* values
%Volume (cm^3^)	–51.5±28.1	–42.5±21.7	.266	–56.4±42.2	–14.3±42.5	**.028** *
%Density	–9.5±12.5	–5.1±9.7	.349	–8.5±11.8	–5.1±12.1	.209
%Mass (g)	–56.9±23.7	–40.0±31.9	.095	–61.8±36.2	–17.9±43.7	**.018** *
Histogram Analysis						
%Kurtosis	–27.7±47.8	–18.4±36.9	.605	–48.0±23.1	1.7±38.6	**.001** *
%Skewness	–26.8±39.8	–22.4±29.1	.760	–34.2±28.9	–1.7±26.9	**.005** *
Texture Analysis						
%Intensity variability	–35.3±31.3	21.6±50.7	**.005** *	92.1±33.3	60.4±81.5	.689
%Size-zone variability	–50.2±23.7	–1.3±40.9	**.003** *	–32.9±21.5	–28.0±32.6	.652

TKI, tyrosine kinase inhibitors; CCRT, concurrent chemoradiation therapy.

Note.—Data are mean ± standard deviation of the percent change n the given parameter.

* *P* <.05.

**Figure 3:**
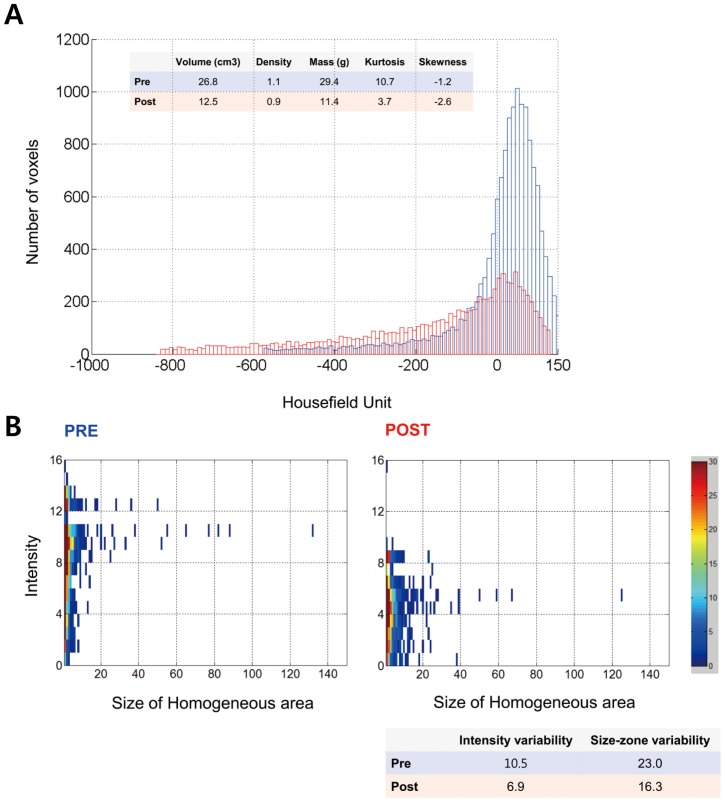
CT images of TKI group and CCRT group. (a) A 56-year-old woman with lung adenocarcinoma who underwent neoadjuvant treatment with EGFR-TKI. Pretreatment CT scans on mediastinal and lung window images show a lobulating sold mass. CT scans obtained after treatment show that the ground-glass opacity component of lesion is remaining on lung window image at the exact area of pretreatment solid component, and reduction of the lesion extent is not significant, (b) A 68-year-old man with lung adenocarcinoma who underwent neoadjuvant treatment with CCRT. Pretreatment CT scans on mediastinal and lung window images show a lobulating sold mass. CT scans obtained after treatment show significant volume reduction of the tumor.

As for histogram analysis, in pathologic responders, the lesion showed negative changes in skewness and kurtosis, indicating platykurtosis and negative skewness (Figure 3). Percent changes of kurtosis and skewness were significantly different between pathologic responders and nonresponders in CCRT group (*p*s = 0.001 and 0.005, respectively), while those were not in TKI group.

On the other hand, in TKI group, intense variability and size-zone variability was significantly decreased in pathologic responders group (*p*s = 0.005 and 0.003, respectively), as compared with nonresponders (Figure 3). These significant differences were not found in CCRT group. Decrease in intense variability and size-zone variability reflects homogeneous change of vascularity [14], [26].

### Logistic Regression Analysis and ROC Analysis

In TKI group, intense variability and size-zone variability were used as input variables for multivariate logistic regression analysis. Multivariate analysis disclosed that intense variability was the sole significant predictor of pathologic response (*p* = 0.028; 95% confidence interval: 1.009, 1.184; adjusted odds ratio, 1.093) (Table 3). ROC analysis showed that the area under the ROC curve (AUC) for intense variability was 0.931 and that the optimal cut-off value of percent change of intense variability for predicting pathologic response was less than –6.9 (sensitivity, 92.3%; specificity, 80%).

**Table 3:** Logistic regression analysis of CT variables for predicting pathologic response.

	Variables	Odds ratio	95% CI	P value
**TKI group**	Intensity variability	**1.093**	**1.009, 1.184**	**0.028**
	Size-zone variability	1.026	0.980, 1.075	0.270
**CCRT group**	Tumor volume (cm^3^)	1.188	0.441, 3.202	0.733
	Tumor mass (g)	0.855	0.320, 2.286	0.756
	Kurtosis	**1.092**	**1.028, 1.160**	**0.004**
	Skewness	1.001	0.919, 1.090	0.982

TKI, tyrosine kinase inhibitors; CCRT, concurrent chemoradiation therapy; AUC, area under the curve; CI, confidence interval.

In CCRT group, tumor volume, mass, kurtosis, and skewness were used as input variables for multivariate logistic regression analysis. Multivariate analysis displayed that kurtosis was the sole significant predictor of pathologic response (*p* = 0.009; 95% confidence interval: 1.026, 1.195; adjusted odds ratio, 1.107) (Table 3). ROC analysis showed that the AUC for kurtosis was 0.943 and that the optimal cut-off value of percent change of kurtosis for predicting pathologic response was less than –23 (sensitivity, 87.5%; specificity, 84.3%).

## Discussion

Our study showed various imaging-based quantitative assessment methods worked differently with different therapeutic regimens, particularly of EGFR-TKI setting and CCRT setting. We found tumor volume, mass, kurtosis, and skewness were all significant predictors of pathologic response in CCRT group in univariate analysis. Using multivariate analysis, kurtosis was found to be independent predictor. On the other hand, in TKI group, heterogeneity from texture analysis was an independent predictor for pathologic response. Our results suggest the possible value of adapting various combination of imaging parameters for response evaluation beyond size-based measurement according to the different treatment modality used. Since there is raising request for personalized and optimized therapy, an advanced diagnostic tool to predict treatment response more accurately is required.

Prognostic factors are an essential requirement in the management of NSCLC and this is reflected in recent changes in the TNM staging [32], where each sub-group is indicative of outcome. However, despite such refinements, there remains uncertainty and more predictive data are required. This is particularly true in Stage 3A disease where outcome remains variable [33]. Moreover, the selection and benefit of surgical patients for neo-adjuvant/ adjuvant treatment is also unclear [34], [35]. It is important that the possible survival benefits of chemoradiation should be balanced by the adverse effects of toxicity to the patient [36]. Through the early identification of patients who will not get a clinical benefit from treatment, patients could be saved from drug toxicity and they could be switched to an alternative treatment earlier. Moreover, there are potential cost savings if drugs are used in only those patients who will benefit from them [14]. Recent additional option of targeted agents makes the situation more complicated. Imaging-defined response assessment (RECIST) is a cornerstone of modern oncologic practice; however, it is limited in a number of targeted therapies, including treatment with TKIs. In a recent study evaluating tumor response to neoadjuvant erlotinib in 60 patients with NSCLC, radiologic response by RECIST was observed in only 5% of the patients, while pathologic response was shown in 23% of the patients [10]. Furthermore, as our figure 2A, the ground-glass opacity component of the tumor may remain at the exact area of pretreatment solid component of the tumor, where volume reduction of the lesion is not significant [19], Therefore, even near complete pathologic response was not predicted by CT-driven RECIST, suggesting that RECIST was suboptimal for short-term radiologic response evaluation. This is because apoptosis, transition of necrosis to fibrosis, or lymphocytic and granulomatous reactions may not result in early decrease in tumor size [10]. These results support the need for development of an optimal imaging parameter for early response evaluation and accurate prediction. In that regard, quantitative CT analysis may be emerging as a potential tool for doing this task.

In the CCRT group, volume decrease was significantly greater in the pathologic responder group, with mean volume reduction of 56.4%. Early volume decrease is achieved by potent locoregional control with cytotoxic effect of irradiation. Volume reduction rate in our study correlates with the results of previous studies [3].

Moreover, skewness and kurtosis could predict pathologic response in the CCRT group. Two values all decreased greater in the pathologic responders, as compared in the nonresponders. Decreased skewness or positive skewness reflects decreased enhancement, which may result from decreased neovascularization.

Meanwhile, CT variables reflecting tumoral heterogeneity were significant prognostic factors for pathologic response in TKI group. A previous study assessing tumor heterogeneity using CT texture in NSCLC patients reported poorer survival in patients who had heterogeneous tumors with low uniformity values [21]. In addition, Tixier et al. analyzed textural features on FDG-PET images in patients with esophageal cancer who underwent concomitant chemoradiation therapy, and found textural features can predict responders (complete and partial) better than can SUV [26]. In their study regional tumor heterogeneity represented by intensity and size-zone variability was a significant predictor of response, rather than global measurement including SUV. Similar results were found in our study, in which intensity variability was a significant independent predictor for pathologic response in patients treated with neoadjuvant TKI agents. Significantly decreased intensity variability and size-zone variability in the pathologic responder group reflects increased tumoral homogeneity, including homogeneous change of vascularity. However, these texture parameters were not helpful in predicting response in the CCRT group. Such reasoning may be explained in part by the difference in pharmagophysiologic reactions according different treatment regimens.

Some authors explain potential significance of tumoral heterogeneity by correlation with genomic heterogeneity [36]. It has recently been shown that a genomic heterogeneity exists within tumors, and this observation has significant implications for Darwinist theories of tumor resistance [37]. Whether textural heterogeneity on imaging relates to underlying genomics would be important to investigate. Given the challenges and expense of measuring tumor genomic signatures, then imaging may be more viable option. Another possible meaning is the possible relationship between tumor heterogeneity and hypoxia. It has been recently shown that tumor textural analysis was associated with tumor hypoxia on histological examinations from NSCLC patients who were administered intravenous pimonidazole prior to surgery [38]. Hypoxia is a recognized marker of poor outcome, and as such, a positive relationship between tumor hypoxia and tumor heterogeneity would be biologically consistent. Additional potential merit of texture analysis is that texture methods can quantify the spatial variations in parametric maps, not the absolute values of the maps. Therefore, texture analysis can provide additional and independent information compared to histogram-based measures of parametric maps [39].

A potential limitation of our study is its retrospective design. However, the majority of baseline characteristics did not differ between the two study populations. Even though there was considerable difference in gender distribution and smoking habit, this occurred because never smoker females were enrolled in the neoadjuvant TKI agent treatment arm, in the clinical setting. A relatively small number of patients were included in our study. Further studies with a larger sample size are necessary for validation of our results. Since tumor regions of interest (ROI) were drawn manually by a single operator, inter- and intra-observer variation may be present. Assistance of an automated software tool could improve this limitation, and furthermore help decrease the time consumed in the tedious work. Despite limitations in our study, it presented novel methodologies that went beyond the traditional size-based analytical method, to evaluate treatment outcomes in NSCLC patients. Our study provides a proof-of-concept that a multiparametric image-feature-based approach holds promise in planning individualized treatment, and that prospective clinical trials may be warranted to better understand the extent of this approach [5].

In conclusion, quantitative CT variables including histogram analysis or texture analysis has potential as a predictive biomarker, and it may better reflect treatment response than standard response criteria based on size change. Also, different combinations of imaging parameters may be correlated to physiologic processes occurring after various different treatment modalities.

## Supporting Information

File S1 (DOCX)
