# Supplementary material for: Quantitative CT Variables Enabling Response Prediction in Neoadjuvant Therapy with EGFR-TKIs: Are They Different from Those in Neoadjuvant Concurrent Chemoradiotherapy?
Source: PLoS One. 2014 Feb 26;9(2):e88598. doi: 10.1371/journal.pone.0088598 (PMC3935840; doi:10.1371/journal.pone.0088598)
Supplement: File S1 — (DOCX) [file pone.0088598.s001.docx]

***Imaging protocol***

In all patients, baseline contrast-enhanced CT before treatment commencement, and follow up contrast-enhanced CT after 6 weeks of neoadjuvant treatment were performed. In all CT examinations, enhanced CT scans were obtained with a 64-detector (LightSpeed VCT XT, GE Healthcare, WI) row scanner. Helical CT scans (125 mA, 120 kVp, beam width of 10–20 mm, beam pitch of 1.375–1.5) were obtained from the lung apices to the level of the middle portion of both kidneys immediately after the completion of intravenous contrast medium injection. An amount of 1.5 mL/Kg (body weight) of Iomeron 300 (Iomeprol, 300 mg iodine/mL; Bracco; Milan, Italy) was injected at an infusion rate of 3 mL/s by using a power injector (MCT Plus; Medrad; Pittsburgh, PA) in all patients. The image data were reconstructed with a section thickness of 2.5 mm. All imaging data were reconstructed by using soft-tissue algorithms. CT data were sent directly to a picture archiving and communication system (Path-Speed or Centricity 2.0; GE Healthcare, Mt. Prospect, Ill). The monitors were used to view both mediastinal (width, 400 HU; level, 20 HU) and lung (width, 1500 HU; level, 2700 HU) window images.

***Image data analysis***

A thoracic radiologist (Y.C., with 4 years of experience in thoracic CT interpretation) who was unaware of other patient data evaluated the acquired images semiquantitatively.

Tumors were segmented by drawing a region of interest (ROI) covering as large an area as possible of the whole tumor. An ROI was drawn freehand around the tumor by using an electronic cursor and mouse. This process was repeated for each contiguous transverse level, until the entire tumor had been covered. Next, voxel-based CT numbers were collected from lesion segmentations.

For tumor density and volume, the computer automatically calculated the density (g/cm^3^) from mean attenuation of total voxels and volume (cm^3^) by multiplying the number of voxels by the unit volume of a voxel [[1](#_ENREF_1)]. Tumor mass (in grams) was calculated by multiplying tumor volume (in cubic centimeters) by mean tumor density [[2](#_ENREF_2)]. Next, a spreadsheet of all of the values was created, which was used to compute histogram distribution parameters of kurtosis and skewness [[3](#_ENREF_3)]. The skewness and kurtosis were computed from the segmented tumor region. The skewness is the third moment of the intensity and used to measure the extent to which a probability distribution of a random variable leans to one side of the mean. The kurtosis is the fourth moment of the intensity and used to measure the peakedness of the probability distribution of random variable [[4](#_ENREF_4)]. An increase in skewness indicated that the skewness values became more positive at the follow-up CT imaging study compared with the baseline. A decrease in skewness meant the values became more negative. In a similar manner, an increase in kurtosis indicated that the kurtosis values became more leptokurtic at the follow-up CT imaging study compared with the baseline CT imaging. A decrease in kurtosis means the values became more platykurtic. On histograms, skewness represented the distribution pattern of CT attenuation values; negative and positive skewness indicated that the data were more spread to the left and right of the mean, respectively. Kurtosis represented the position of peak height that indicates CT attenuation value of the maximum number of voxels, with leptokurtic indicated by a sharper peak and platykurtic indicated by a flatter peak (Figure 1).

Texture analysis was performed by a radiology physicist (J.H.K.) with four years of experience in radiology physics, who was blinded to the clinical outcome. Voxel values within the segmented tumors were resampled to yield 16 of discrete values in order to reduce the noise in image and to normalize the intensity across subjects by clustering voxels with similar intensities [[5](#_ENREF_5)]. From the discrete tumor images (16 gray levels), the gray level size zone matrix was computed. The value of the matrix’s (m, n) is defined by the number of homogenous regions given the homogeneous tumor size (n) to their intensity (m). For example, ‘Matrix’s (3, 5) is 6.’ means that there are 6 homogenous regions, in which the grey level of each cluster is 3, and the size of each cluster is 5 voxels. This gray level size zone matrix was used to compute the variability in the size and the intensity of homogeneous tumor regions [[5](#_ENREF_5),[6](#_ENREF_6)].

***Pathologic evaluation of treatment response***

Pathologic response was used as the reference standard of therapeutic response. Resected tissues from primary tumors were formalin ﬁxed and parafﬁn embedded. An experienced lung pathologist (J.H. with 20 years of experience in lung pathology) retrospectively interpreted entire tissue sections sliced at 5- to 10-mm intervals and measured the proportion (%) of viable tumor cells in the primary tumor of the resected surgical specimens [[7](#_ENREF_7)]. Tumor regression was scored as ’pathologic response’ if more than 50% necrosis was present with morphologic signs of therapy-induced regression or ‘no response’ when either 0%–50% necrosis or necrosis that could not be attributed to the therapy effect was seen [[8](#_ENREF_8)]. If more than 90% necrosis was present in the resected specimen, tumor regression was deﬁned as ‘near- complete pathologic response’ [[8](#_ENREF_8)].

In addition, comprehensive histologic subtyping was made for the primary tumor in a semi-quantitative manner. The extent of existent tumor histologic subtypes was quantiﬁed to the nearest 5% level [[9](#_ENREF_9)]. Then, the single most predominant pattern (the highest percentage of a particular tumor component) was chosen as the histologic subtype of the tumor. Tumors were classiﬁed according to the IASLC/ATS/ERS classiﬁcation as: preinvasive adenocarcinomas including adenocarcinoma-in situ (AIS); minimally invasive adenocarcinoma (MIA); and invasive adenocarcinomas, which were further subdivided into showing lepidic predominant, acinar predominant, papillary predominant, micropapillary predominant, and solid predominant patterns [[9](#_ENREF_9)]. Tumors were stratiﬁed into the following three grades based on histologically predominant subtypes as: low grade (AIS, MIA, and lepidic predominant); intermediate grade (papillary and acinar predominant); and high grade (micropapillary and solid predominant) [[10](#_ENREF_10),[11](#_ENREF_11)]. One pathologist evaluated all the resection specimens and was unaware of other patient data.

*EGFR/KRAS* mutational status had been determined by a pathologist (Y.L.C.) by using polymerase chain reaction (PCR) and a direct DNA sequencing method, as previously described [[12](#_ENREF_12)].

**References**

[1] de Hoop B, Gietema H, van de Vorst S, Murphy K, van Klaveren RJ, Prokop M. Pulmonary ground-glass nodules: increase in mass as an early indicator of growth. Radiology 2010;255:199-206.

[2] Lee HY, Jeong JY, Lee KS, et al. Solitary pulmonary nodular lung adenocarcinoma: correlation of histopathologic scoring and patient survival with imaging biomarkers. Radiology 2012;264:884-93.

[3] Chandarana H, Rosenkrantz AB, Mussi TC, et al. Histogram analysis of whole-lesion enhancement in differentiating clear cell from papillary subtype of renal cell cancer. Radiology 2012;265:790-8.

[4] Groeneveld RA, Meeden G. Measuring Skewness and Kurtosis. J R Stat Soc Series D (The Statistician) 1984;33:391-99.

[5] Tixier F, Le Rest CC, Hatt M, et al. Intratumor heterogeneity characterized by textural features on baseline 18F-FDG PET images predicts response to concomitant radiochemotherapy in esophageal cancer. J Nucl Med 2011;52:369-78.

[6] Thibault G, Fertil B, Navarro C, et al. Texture indexes and gray level size zone matrix: application to cell nuclei classiﬁcation. Pattern Recognition Inf Process 2009:140-45.

[7] Lee HY, Lee HJ, Kim YT, et al. Value of combined interpretation of computed tomography response and positron emission tomography response for prediction of prognosis after neoadjuvant chemotherapy in non-small cell lung cancer. J Thorac Oncol 2010;5:497-503.

[8] Aukema TS, Kappers I, Olmos RA, et al. Is 18F-FDG PET/CT useful for the early prediction of histopathologic response to neoadjuvant erlotinib in patients with non-small cell lung cancer? J Nucl Med 2010;51:1344-8.

[9] Travis WD, Brambilla E, Noguchi M, et al. International association for the study of lung cancer/american thoracic society/european respiratory society international multidisciplinary classification of lung adenocarcinoma. J Thorac Oncol 2011;6:244-85.

[10] Kadota K, Colovos C, Suzuki K, et al. FDG-PET SUVmax combined with IASLC/ATS/ERS histologic classification improves the prognostic stratification of patients with stage I lung adenocarcinoma. Ann Surg Oncol 2012;19:3598-605.

[11] Lee HY, Jeong JY, Lee KS, et al. Histopathology of Lung Adenocarcinoma Based on New IASLC/ATS/ERS Classiﬁcation: Prognostic Stratiﬁcation With Functional and Metabolic Imaging Biomarkers. J Magn Reson Imaging 2013.

[12] Yoon HJ, Lee HY, Lee KS, et al. Repeat biopsy for mutational analysis of non-small cell lung cancers resistant to previous chemotherapy: adequacy and complications. Radiology 2012;265:939-48.
